# Sleep disturbance in Angelman syndrome patients

**DOI:** 10.1186/s13023-024-03154-5

**Published:** 2024-04-05

**Authors:** Song Qu, Junyi Wang, Xingying Guan, Cui Song, Yanyan Wang

**Affiliations:** 1 Department of Medical Genetics, College of Basic Medical Science, Army Medical University (Third Military Medical University), Chongqing, China; 2 Department of Endocrinology and Genetic Metabolism Disease, Children’s Hospital of Chongqing Medical University, National Clinical Research Center for Child Health and Disorders, Ministry of Education Key Laboratory of Child Development and Disorders, Chongqing Key Laboratory of Pediatrics, Chongqing, China

**Keywords:** Angelman syndrome, Sleep disturbance, Imprinting disorder

## Abstract

Angelman syndrome (AS) is a neurodevelopmental disorder caused by abnormal expression of the maternal ubiquitin protein ligase E3A gene (*UBE3A*). As one of the most challenging symptoms and important focuses of new treatment, sleep disturbance is reported to occur in 70–80% of patients with AS and has a serious impact on the lives of patients and their families. Although clinical studies and animal model studies have provided some clues, recent research into sleep disorders in the context of AS is still very limited. It is generally accepted that there is an interaction between neurodevelopment and sleep; however, there is no recognized mechanism for sleep disorders in AS patients. Accordingly, there are no aetiologically specific clinical treatments for AS-related sleep disorders. The most common approaches involve ameliorating symptoms through methods such as behavioural therapy and symptomatic pharmacotherapy. In recent years, preclinical and clinical studies on the targeted treatment of AS have emerged. Although precision therapy for restoring the UBE3A level and the function of its signalling pathways is inevitably hindered by many remaining obstacles, this approach has the potential to address AS-related sleep disturbance.

## Background

Angelman syndrome (AS) is a neurodevelopmental disorder caused by loss of function of the ubiquitin–protein ligase E3A (*UBE3A*) gene, which, in almost all neurons, is expressed only from the maternal chromosome 15. It was first diagnosed by Dr. Harry Angelman in 1965 as 'happy doll syndrome'. He reported similar abnormal behavioural features in three children, including six distinct central nervous system disorders (mental retardation, speech impairment, motor and ataxia abnormalities, microcephaly, atrophy, and ventricular dilatation) and sudden laughter, which was subsequently named AS [1].

AS is a rare neurogenetic disorder with a prevalence of 1 in 20,000 to 1 in 12,000 people [2, 3]. Together, these findings indicate that both paternal *UBE3A* silencing and maternal loss of function contribute to AS. Four genetic mechanisms may contribute to maternal *UBE3A* loss of function: (1) maternal deletion of chromosome 15q11-13 (approximately 65–70% of the AS population); (2) maternal allelic point mutations or small fragment deletions (5–11%); (3) paternal uniparental disomy (3–7%), where two paternal copies of an epigenetically silenced *UBE3A* allele are inherited; and (4) imprinting centre defects (3%).

Sleep disorders are among the most common problems associated with AS. It is estimated that 70–80% of people with AS exhibit sleep disorders [4, 5]. First and most importantly, sleep is closely linked to learning memory and brain development by categorizing and reinforcing newly encoded memories in the absence of constant inputs from external information. Moreover, sleep and quality of life are closely linked, so sleep problems in children with AS have a substantial impact on both patients and their caregivers. Existing treatments can relieve and control only some of the symptoms in a variety of ways, and these approaches include environmental changes or the use of psychotropic medications. The effectiveness of behavioural therapy and symptomatic medication treatment is limited for AS patients, and the underlying mechanisms are unclear. Sleep problems are often difficult to stabilize through these interventions, especially at a young age [6]. With the development of technology, molecular targeted therapies guided by specific genetic changes are becoming increasingly common, and this approach has potential for the treatment of patients with AS [7]. Therefore, this report reviews the characteristics of AS-related sleep disorders, research methods, AS-related neural circuit mechanisms of sleep, and AS-related sleep treatments based on the molecular mechanisms of AS.

## Main text

### Current status of research on sleep disturbance in AS patients

Patients with AS have a reduced need for sleep and suffer from difficulty falling asleep and frequent nighttime awakenings [8]. Recent research on AS-related sleep disorders has been based on two main categories of evidence, clinical studies and animal model studies, both of which provide important information.

#### Evidence from clinical studies

Clinical studies can be divided into survey studies and polysomnography (PSG) studies. Survey studies, which are based on parental and caregiver reports, provide subjective information about mood, sleep patterns, sleep habits and behavioural consequences. According to a caregiver-based telephone interview, 72% of people with AS reported having a 'sleep disorder' that severely affected their lives [9]. Walz et al. used a validated sleep questionnaire to investigate the sleep patterns of 339 patients registered with the AS Foundation, and 48% and 42% of the respondents reported that they had difficulty falling asleep and slept less than their peers, respectively [10]. Although different laboratories have confirmed that AS patients have severe sleep disorders, the findings of different laboratories on AS-related sleep disorders are not entirely consistent. A questionnaire survey of 109 Dutch people revealed that 40% of patients had severe sleep problems, the main symptom of which was nocturnal awakening rather than increased sleep latency [11]. Another clinical review indicated that increased sleep onset latency, shortened sleep duration, and disrupted sleep architecture with frequent nocturnal awakenings were characteristic problems. Furthermore, there is evidence in the literature that AS-related sleep problems do not affect the daytime status of patients [6], and although the daily amount of sleep is significantly less than the average for healthy individuals, it appears to have less impact on their health and behaviour [12, 13].

Several genetic mechanisms impair UBE3A expression, but they differ in how neighbouring genes on chromosome 15 at 15q11–q13 are affected. Several studies have shown a more severe clinical phenotype for AS patients with a deletion than for those without a deletion, and some suggest that larger deletions lead to more severe impairment than smaller deletions [14]. The larger deleted regions included *UBE3A, GABRB3*, *GABRA5*, and *GABRG3*, genes that encode the gamma-aminobutyric acid (GABA) type A receptor subunits β3, α5, and γ3, respectively. Because GABA activation plays an important role in sleep [15], deletion of the *GABRB3-GABRA5-GABRG3* gene cluster likely contributes to a more severe clinical phenotype of sleep problems. GABA is the most abundant inhibitory neurotransmitter in the brain and is implicated in decreasing cortical activation, inducing non-REM sleep and influencing the initiation and maintenance of sleep. An analysis of phenotype and genotype in a large cohort of Chinese children with AS suggested that sleep disturbance was common in AS patients, and the deletion group had a greater incidence of sleep disturbance (89.08%) than the nondeletion group (83.90%) [16]. Joel et al*.* suggested that the *GABRB3-GABRA5-GABRG3* gene cluster causes abnormal theta and beta EEG oscillations that may underlie the more severe clinical phenotype [17]. More systematic quantitative investigations about sleep problem differences between AS genotypes are needed.

Most of these results are based on subjective evidence from naturally short sleepers and lack objective quantification. Therefore, research on sleep in AS patients may provide important information about the nature of sleep requirements and related health problems and reveal new mechanisms of sleep regulation, but an objective and valid approach is needed to provide an in-depth understanding of sleep disorders in AS patients.

Importantly, PSG based on electroencephalogram (EEG) data can be used as an objective potential method for studying sleep disorders in AS patients. In a retrospective study, den Bakker and his colleagues identified two abnormal EEG features of sleep disorders in children with AS: increased gamma coherence and reduced numbers of sleep spindle waves [18]. Frequent changes observed during the interictal periods in AS include theta and delta waves intermixed with sharp or spike discharges, revealing the characteristic appearance of 'notched delta waves' [19, 20]. However, the extent to which these EEG abnormalities manifest in sleep disorders in patients with AS is unknown, and these data were recorded during waking or spontaneous sleep, while the incidence of abnormalities in specific sleep/wake states has not been reported [20, 21]. Thus, state-specific analysis is essential for determining the extent to which EEG abnormalities are associated with sleep disorders in patients with AS. The PSG can provide an objective indicator of sleep state, quantity, quality and potential diagnostic criteria, and the use of PSG can achieve the goal of monitoring a patient's sleep/wake state in real time because the EEG is recorded. The PSG uses EEG and additional measurements (e.g., electromyography (EMG)) to objectively assess the degree of sleep and wakefulness with far better accuracy than behavioural analysis. Thus, when EEG data are combined with other PSG data, an objective analysis of sleep in patients with AS can be performed. State-specific studies of patients with AS have revealed that abnormal EEG results are prevalent in these patients during sleep [21, 22]. In a PSG study of AS patients, multiple sleep parameters indicated decreased sleep efficiency. Patients with AS experienced a nearly twofold increase in the number of transitions between sleep states, a fourfold increase in the frequency of awakenings (i.e., sleep fragmentation) and a 50% reduction in the time spent in the deepest stage of nonrapid eye movement (NREM) sleep, all of which indicate reduced sleep quality and sleep efficiency during the night in patients with AS [23, 24]. Differences in the number and duration of spindle waves during the NREM sleep stages have also been reported recently. In one study, approximately half of the children with AS had significantly reduced numbers of sleep spindle waves on EEG. This spindle wave activity in the 11–16 Hz range occurs during NREM sleep and is associated with memory consolidation, and sleep is essential for cognition, suggesting a potential direct relationship between poor sleep quality and cognitive deficits in patients with AS [18]. Despite the deficits in NREM sleep quality, there is no indication of a reduction in the total amount of NREM sleep in people with AS. This finding somewhat contradicts subjective parental or caregiver reports of overall sleep duration. However, although the extent of sleep defects in people with AS is unclear, there is no doubt that their sleep quality is significantly impaired. Studies have shown that patients with AS have significantly less rapid eye movement (REM) sleep than healthy control individuals do, which may be a direct result of poor NREM sleep in patients with AS, as they may not be able to effectively work through the 3 stages of NREM to achieve REM sleep [23, 24]. Thus, the objective PSG findings are consistent with caregivers' reports of poor nighttime sleep in AS patients. However, the slight inconsistency in overall sleep duration makes it difficult to predict the underlying cause of this sleep disorder.

Only a few studies assessing patients with AS have involved survey-based and PSG-based methods, but no new PSG studies on patients with AS have been published since the descriptive review by Pelc et al. [6]. Overall, more rigorous sleep research methods are needed for the study of AS-related sleep disorders, and in general, standardization of sleep studies in patients with AS is essential for the development of new research directions [6]. In turn, the standardization and improved reproducibility of experimental studies cannot be achieved without the use of experimental animal models.

#### Evidence from animal model studies

In addition to clinical studies, animal model studies provide detailed sleep indicators and analyses; these data are not available for humans and can be used to assess the safety effectiveness of therapeutic drugs and other treatment modalities. Clinical studies on AS-related features of sleep are limited, but animal models provide many opportunities to determine the effects of *Ube3a* deficiency on circadian rhythms and the sleep system [25].

To date, several AS models have been developed after the identification of the *Ube3a* gene as the major contributor to AS and its inheritance. These models can recapitulate some neurological phenotypes present in AS patients, including motor dysfunction, dyskinesia, epilepsy, learning disabilities, and abnormal electroencephalographic patterns [26–28] (see Table 1).

**Table 1:** The typical AS models

	**Method**	**Reference**
***Ud5***^***m−/p*+**^** mice**	Deleting exon 5 (290 nt) from the transcriptional start site in the maternal *Ube3a* allele	Jiang et al*.* [29]
**Large deletion mice**	Deleting ~ 1.6 Mb chromosome including *Ube3a* gene	Jiang et al*.* [30]
***Ud6***^***m−/p*+**^** mice**	A larger deletion of exon 6 (1247 nt) in the *Ube3a* gene than *Ud5*^*m−/p*+^ mice	Shi et al*.* [25]
***Ube3a***^***tm1.1Bdph***^** mice**	*Ube3a*^*mflox/p*+^ transgenic mice received tamoxifen to induce Cre-mediated deletion of the* Ube3a*	Monica et al*.* [31]
***Ube3a***^***LacZ***^** mice**	Replacing part of exon 15 and full exon 16 of *Ube3a* gene with the *β-galactosidase* (*LacZ*) transcriptional reporter	Miura et al*.* [32]
***Ube3a-YFP* reporter mice**	Infusing *YFP* after *Ube3a*	Dindot et al*.* [33]
***Ube3a***^***IC−KO***^** mice**	Deleting the AS imprinting center (IC)	Lewis et al*.* [34]

Table 1 AS mouse models and the generating methods. These models are developed to explore the underlying mechanism of AS and verify the effectiveness of therapy

AS-large deletion mice are generally considered to better reflect AS in humans than *Ube3a*^*m−/p*+^ mice because most patients with AS (∼75%) have maternal deletions of chromosome 15q11-13, including *Ube3a, Atp10a, and Gabrb3* [30]. The *Ube3a*^*m−/p*+^ mice are specific for the *Ube3a* gene, which is primarily responsible for AS and therefore allows the precise association of aberrant phenotypes with a reduced dosage of *Ube3a* [29]. Both of these indices were used to assess sleep disturbance. For example, using Jiang and Beaudet's mouse model of AS, many laboratories have recorded activity and circadian rhythm patterns in mice and reported many features of sleep associated with AS, including sleep defects, reduced activity, prolonged circadian cycles, reduced adaptability to rhythm changes, and delayed response to sleep deprivation [35, 36]. However, there is still disagreement among the findings regarding the sleep characteristics of mouse models. In a study by Shi et al., neuronal imprinting of *Ube3a* led to reduced activity and prolonged circadian rhythm cycles in two mouse models (AS-large deletion and *Ube3a*^*m−/p*+^ mice) and consequently to phase delays, which could explain the short sleep duration and increased sleep onset latency in subjects with AS [26]. However, the AS *Ube3a*^*m−/p*+^ mice in that study [29] maintained a relatively normal circadian rhythm pattern and showed enhanced activity when awake and skipped the mouse-specific nap phase [37]. There are still some problems to be solved in different mouse models. In a review, the current controversy was analysed, and it was concluded that the diminished robustness of circadian rhythms and the reduced ability to accumulate sleep pressure were present in AS mice [37]. However, these differences in sleep analysis results between laboratories are influenced by many factors, such as the methodological approach used for sleep recording, the background of the mouse line, the age of the mice and the EEG acquisition technique used [35, 36]. This is one of the issues that needs to be addressed in ongoing AS sleep studies to control for confounding factors, standardize the sleep study process and produce comparable results between laboratories.

Recent progress in the study of sleep disorders in AS has been slow, mainly because there are few sleep research methods available for providing an in-depth characterization and analysis of sleep disorders. There are many mouse models, but none of them can completely reproduce the phenotype of AS-related sleep disorders or be used to establish a consistent conclusion. Moreover, the ubiquitination substrates of UBE3A are universal, which challenges the study of the signalling pathways and mechanisms of AS-related sleep disorders.

### The mechanism of sleep disorders in AS

AS results from loss of function of the imprinted *UBE3A* gene. The UBE3A protein was originally described as a link between p53 and the E6 oncoproteins of various human papillomavirus types [38]. The E3 ubiquitin ligase binds to p53 and degrades it through the ubiquitin–protein hydrolysis system. The gene is approximately 120 kb in length and encodes a variety of isoforms that may differ in substrate specificity, function, and cell localization patterns [39, 40]. UBE3A is a member of the HECT family of enzymes that plays an important role in transferring activated ubiquitin to proteins and degrading them through the protein hydrolysis system [41]. UBE3A is also a nonspecific transcriptional coactivator of the nuclear hormone receptor and is not dependent on its ligase activity, as mutations affecting E6-AP activity do not alter its coactivation capacity [42, 43]. Although the function of UBE3A as a ubiquitin ligase protein and transcriptional coactivator is clear, the exact mechanism by which it contributes to AS through the loss of function of the maternal allele in AS is unclear. *UBE3A* is located at chromosomal region 15q11-13 and exhibits biallelic expression throughout most of the body, but only the maternal allele is expressed in neurons due to imprinting [44]. Paternal *UBE3A* is silenced by the long noncoding RNA (> 600 kb) antisense transcript *UBE3A-ATS* [45]. *UBE3A* deletion results in a decrease in E3 ubiquitin ligase levels, a decrease in the ability of ubiquitin proteins to transfer substrates, and a consequent decrease in the ability of the proteasome to degrade or regulate cellular functions.

**Fig. 1:**
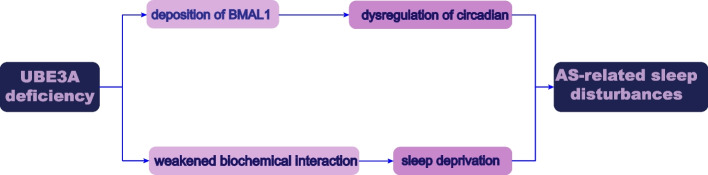
The mechanism of sleep disturbances in AS

During sleep, the myriad neural networks involved in memory processing are endogenously activated. The use of noninvasive surface electrodes or intracranial electrodes makes it possible to record electrical waves during sleep. Among the captured electrical fluctuations, several patterns, including oscillations, transient potentials with recognizable waveforms, and spike activity patterns, are used to clarify the processes that occur in the brain. For example, during sleep, slow cortical oscillations and REM sleep theta oscillations combine to improve memory. Consistently, studies have shown that circadian rhythms influence hippocampal plasticity and cortical development. The hippocampus is often associated with the formation of new associative memories, the storage of memories independently, the retrieval of memories from partial cues, and the flexible application of stored memories to new situations [46]. With sleep deprivation, hippocampal plasticity decreases, hippocampal and cortical patterns are difficult to coordinate during NREM sleep, and theta oscillation regulation in the hippocampus has difficulty influencing learning and memory consolidation [47]. Mutations in *UBE3A* lead to a decrease in BMAL1 protease inhibitor levels and the deposition of BMAL1, resulting in dysregulation of circadian rhythms in AS patients. Furthermore, mutations in *UBE3A* lead to a weakened biochemical interaction between mGluR5s and postsynaptic HOMER1A, which is associated with sleep deprivation (SD), resulting in sleep stress in AS patients. In turn, sleep disturbance further affects the neurodevelopment of AS patients through synaptic excitotoxicity and dysregulation of metabolic pathways, thereby exacerbating neurodevelopmental abnormalities in AS patients [48]. In conclusion, the absence of UBE3A leads to circadian dysregulation and sleep deprivation, which in turn affects hippocampal neurons, resulting in sleep disturbance, reduced learning and memory capacity, and neurodevelopmental impairment. Therefore, targeted treatment of AS patients to restore neuronal *UBE3A* expression may effectively improve sleep quality, cognition, and quality of life (Fig. 1).

### Therapeutic treatment of sleep disorders in AS patients

There is no aetiological therapy for patients with AS. Behavioural therapies and common medications are often used to ameliorate symptoms in the treatment of AS-related sleep disorders. Investigations of targeted therapies based on the aetiological mechanisms of sleep in AS patients are still in the preclinical or clinical stages, and these treatments need to be further explored to identify safer and more effective treatments.

#### Behavioural therapy

Behavioural therapy is now recognized as the 'first-line' treatment for sleep disorders in children, with numerous studies showing that this approach produces sustained changes in more than 80% of children [49, 50]. In behavioural therapy, parents are instructed on how to participate in and implement these therapies to model healthy and good sleep habits in their children [51]. Essential elements of quality sleep interventions include teaching parents how to create a quality sleep environment, adjusting sleep–wake schedules to consolidate sleep and managing parent‒child interactions to reinforce appropriate bedtime behaviour and promote independent sleep initiation [52]. The effectiveness of behavioural approaches is partially supported empirically in the sleep literature, and behavioural approaches are recommended for the management of sleep problems associated with AS [6]. However, few parents of children with AS have accepted such advice for the treatment of their sleep problems. This may be due, in part, to concerns about the appropriateness of behavioural treatments for sleep problems in children with AS. That is, some providers and parents may question whether behavioural interventions can address sleep problems caused by structural abnormalities or imbalances in cortical interactions [6, 10]. Furthermore, patients and their families may be reluctant to accept behavioural therapies without sufficient empirical support. Indeed, practitioners and researchers agree that additional research on behavioural approaches to managing sleep problems in children with AS is needed [6, 11]. To date, only one case study evaluating behavioural therapy for children with AS has been published. Summers et al. treated a 9-year-old boy with AS who slept less than 2 h per night [53]. These behavioural interventions included limiting daytime sleep, setting a consistent sleep schedule, and reducing adult–child interactions at night. The intervention included the use of the preexisting drug Benadryl. Additionally, in-home behavioural treatment was started only after 55 days of inpatient treatment. Therefore, the independent effects of behavioural interventions or the ability of parents to implement the interventions were never assessed, although treatment was effective and the child's sleep time increased to more than eight hours per night [53]. Therefore, although behavioural sleep interventions are often recommended for children with AS, there are no well-controlled empirical studies on the behavioural treatment of sleep problems in children with AS.

#### Symptomatic treatment with medication

A literature review characterized many medications (melatonin receptor agonists, antidepressants (mirtazapine), antihistamines (norepinephrine or diphenhydramine), benzodiazepines, orexin antagonists, antipsychotics, anticonvulsants, and 'z-drugs') from many different classes and presented the available evidence regarding their efficacy and safety as a basis for clinical decision making [54]. To date, no systematic evaluation of the management of AS-related sleep disorders has been performed. Moreover, few AS patients with sleep disorders have undergone randomized, double-blind, placebo-controlled trials [55].

##### Melatonin receptor agonists

Melatonin and ramelteon are two melatonin receptor agonists used to treat sleep disorders. Literature data on AS-related sleep disorders report the predominant use of melatonin. Melatonin is a hormone that is taken up by many individuals with sleep disorders. Normally, it is released by the pineal gland during the dark period of the day. It binds predominantly to the MT1 and MT2 receptors, though the mechanism by which this binding might enhance sleep is poorly understood [56]. A randomized placebo-controlled trial and a small cohort prospective study reported that the use of melatonin was effective for treating sleep disorders in patients with AS [57, 58]. Melatonin appears to improve sleep parameters and daytime behaviour in patients with autism spectrum disorders. However, the results of these studies were not entirely reliable because only subjective scales or surveys were used to measure sleep. Moreover, none of the studies compared the efficacy of melatonin with that of other treatments [57–59].

##### Antidepressants

Several medications were originally developed for the treatment of major depressive disorder and are commonly used for treating sleep disorders. These agents may enhance sleep effects by blocking the receptors for neurotransmitters that are wake enhancing [60]. Mirtazapine is one of the antidepressants that is most commonly used to treat sleep disorders. It has been reported to have significant efficacy in terms of improving sleep duration, reducing night waking and reducing sleep latency in AS patients [61]. However, the methods applied in these studies are unreliable and include inconsistent use of objective outcome measures. Therefore, additional controlled studies are needed to fully corroborate the value of mirtazapine in the treatment of AS-related sleep disorders.

##### Antihistamines

Several antihistamines that are commonly used for treating sleep disorders include diphenhydramine, doxylamine, doxepin and niaprazine, which are involved in many sleep disorder therapies. All of these agents have either H1 antagonism or clinically relevant M1 muscarinic cholinergic antagonism [54]. Niaprazine appears to be very effective for treating AS-related sleep disorders based on clinical experience [62]. It is a histamine H_1_-receptor antagonist that has an antihistaminic effect. Niaprazine, which has been used in people with behaviour and sleep disorders, differs from other antihistamines, particularly because of its marked sedative properties. Unfortunately, its use is restricted in many countries due to its scarcity [63].

In brief, only a small number of these medications from several different classes have been evaluated in patients with AS-related sleep disorders. The data are limited by the small sample size and lack of replication. In addition, there is very limited evidence regarding the use of other medications, such as antipsychotics, anticonvulsants, orexin antagonists, benzodiazepines, and 'z-drugs', in patients with AS. Therefore, additional studies are needed to confirm the effectiveness and usefulness of other medications. Interestingly, a small study suggested that iron deficiency may be associated with sleep difficulties in patients with AS. Iron supplementation may modestly improve sleep quality in the AS patient population [64].

#### Precise treatment

Precision therapy offers hope for the treatment of many genetic disorders that are difficult to treat with conventional therapies, and promising advances have been made in translational research on AS. Several strategies are undergoing preclinical and clinical development for the treatment of AS. The introduction of a functional *UBE3A* copy or the reactivation of a silenced but still functional *UBE3A* copy on the paternal allele are the most promising therapeutic strategies [7, 27]. Data from phase 1–2 clinical trials of antisense oligonucleotide (ASO) compounds have shown encouraging results, with ASO treatment achieving specific reductions in *UBE3A-ATS* levels in neurons in vivo and in vitro and restoring *UBE3A*-mRNA and protein products in the neurons of AS patients [7]. In another study, activation of silent paternal *Ube3a* using topotecan, which is a regulator of the core clock protein BMAL1, restored the circadian cycle of neurons in brain slices from AS mice [26, 65]. Treatment of sleep in patients with AS may be more prospective from an aetiological point of view, but there are still some barriers, such as the invasive and damaging nature of ASO treatment for children. Topotecan, an anticancer agent, has severe hepatic and renal toxicity. Therefore, the pathogenesis of AS needs to be further explored, and technical issues need to be solved, including the determination of the optimal treatment window, assessment of the degree of harm caused by invasive treatment and evaluation of drug safety [7].

## Conclusions

AS is a rare neurodevelopmental disorder caused by the loss of function of the maternally expressed *UBE3A* gene in the brain. Sleep problems seriously affect patients’ daily lives and cause great disturbance to their families. Although research on the sleep phenotype and its aetiological mechanism in AS has been ongoing, this research is limited to objective and accurate sleep quantification techniques and other methods. To date, there is no consistent conclusion from research on AS-related sleep disturbances in patients and mouse models. This has impeded progress in understanding the mechanism of sleep problems and their treatment. Multimodal intervention, including behavioural rehabilitation and symptomatic pharmacotherapy combined with personalized precision therapy, is an efficacious therapeutic option for improving the quality of sleep in patients with AS. However, to date, there are no clinically available disease-modifying therapies for AS, and behavioural and pharmacological interventions are aimed only at alleviating the severe sleep disorder phenotype; thus, accelerating the development of safe, stable and feasible precision therapies for AS is highly important.

AS-related sleep disturbances results from loss of function of UBE3A gene. *UBE3A* deficiency leads to a decrease in BMAL1 protease inhibitor levels and the deposition of BMAL1, resulting in dysregulation of circadian rhythms in AS patients. Furthermore, it leads to a weakened biochemical interaction between mGluR5s and postsynaptic HOMER1A, which is associated with sleep deprivation, causing sleep stress in AS patients. In turn, sleep disturbance will further affect the neurodevelopment of AS patients through synaptic excitotoxicity and dysregulation of metabolic pathways, thereby exacerbating neurodevelopmental abnormalities in AS patients.

## Data Availability

We do not generate any datasets, because our work proceeds within a theoretical approach. One can obtain the relevant materials from the references below.

## Abbreviations

AS: Angelman syndrome
UBE3A: Ubiquitin protein ligase E3A gene
PSG: Polysomnography
EEG: Electroencephalogram
EMG: Electromyography
NREM: Nonrapid eye movement
REM: Rapid eye movement
SCN: Suprachiasmatic nucleus
ASO: Antisense oligonucleotide
